# Investigation on the Electrical Conductivity of Graphene/Cement Composites by Alternating Current Method

**DOI:** 10.3390/ma16041436

**Published:** 2023-02-08

**Authors:** Ming Jin, Wenwei Li, Yuefeng Ma, Haoyu Zeng, Minghui Huang, Chao Lu, Guo Yang

**Affiliations:** 1 School of Material Science and Engineering, Southeast University, Nanjing 211189, China; 2 Division of Science and Technology Management, China Three Gorges Corporation, Wuhan 430010, China; 3 College of Materials Science and Engineering, Chongqing University, Chongqing 400045, China; 4 China Three Gorges Construction Engineering Corporation, Chengdu 610095, China

**Keywords:** graphene, cement paste, electrical properties, AC technique

## Abstract

This paper is concerned with an analysis of the electrical conductivity of graphene/cement composites by means of DC (direct current) and AC (alternating current) techniques. Moreover, the micrograph and element composition of composites have been characterized through SEM (scanning electron microscopy) and EDS (energy-dispersive spectrometers) techniques, respectively. Results revealed that a percolation transition region Φ2–Φ1 (Φ2 and Φ1 values are determined as 0.8% and 1.8%, respectively) can be observed in the S-shaped curve. In addition, the logistic model has been recommended to characterize the relationship between the conductivity and the graphene concentration, which ranged from 0.001% to 2.5%. The micrographs obtained by SEM technique clearly indicate a complete conductive network as well as agglomeration of graphene slices when the graphene content reaches the threshold value. Furthermore, graphene slices can be distinguished from the cement hydration products by means of the analysis of element composition obtained through the EDS technique. It is promising to apply the graphene/cement composites as intelligent materials.

## 1. Introduction

Electrically conductive cement-based materials have been regarded as one category of functional cement-based materials. The electrical conductivity of plain cement paste under dry condition usually ranges from 8.7 × 10^−7^ Ω^−1^·cm^−1^ to 1.5 × 10^−6^ Ω^−1^·cm^−1^ [1]. This clearly indicated that the cement paste was a poor electrical conductor. This poor conductivity property restricted its use as a functional material, which required high electrical conductivity in certain conditions such as structural health monitoring [2,3,4]. Hence, improvement in the conductive performance of cement-based materials has attracted much attention.

Chung [5] compared the effectiveness of several conductive admixtures (i.e., steel fiber, carbon filament, carbon fiber, graphite powder, and coke powder) at similar fractions in cement paste. They reported that stainless steel fiber with a diameter of 8 μm was the most effective for increasing the electrical conductivity among these carbon and steel admixtures. The conductivity of the cement paste with the addition of steel fiber exceeded 0.06 Ω^−1^·cm^−1^. Much attention has been paid to improvement of the conductivity of cement paste by means of graphite [6], carbon fiber [7,8], and carbon nanotube [9,10]. Chen et al. [11] found out that the conductivity for carbon-fiber-reinforced cement-based material could reach 0.005 Ω^−1^·cm^−1^. In addition, Xu et al. [8] carried out research on the nonlinear electrical behavior of carbon fiber/cement composites by the application of tunneling influence theory and Ohm’s law. As a result of recent advances in materials science and nanotechnology, graphene has emerged as a revolutionary material. Graphene has excellent properties, including high intrinsic mobility (200,000 cm^2^ v^−1^ s^−1^) and large theoretical specific surface area (2630 m^2^ g^−1^) [12]. Moreover, graphene also has good physical properties, and gaphene oxide has been used to improve the mechanical performances of cement-based materials [13,14,15]. Therefore, adding graphene to cement paste would significantly increase its conductivity. Although improvement in the conductivity of cement-based materials by graphene has been reported in some research [2,16,17,18,19], the underlying influencing mechanism of graphene on the electrical conductivity of cement-based materials needs to be further clarified.

Concerning the relationship between the conductivity and the content of conductive materials, the percolation transition zone has been widely found in the curve, which could be explained by the application of percolation theory. Based on this, a percolation equation has been used to characterize the relationship between conductivity and conductive material content [20]. However, this equation had some significant disadvantages; for example, it is difficult to determine the $\varphi_{c}$ value. To make matters worse, graphene contents lower than the $\varphi_{c}$ value were not included in the domain of the definition. Hence, there was an urgent need to find out a new model or equation by which the comprehensive characterization of the relationship between the conductivity and the whole contents could be realized.

In this study, the conductivity of graphene/cement composites was investigated by direct current (DC) and alternating current (AC) techniques. In addition, both logistic model and percolation theory were used to describe the relationship between the conductivity of cement/graphene composites and graphene concentration. The micro-morphology and element compositions of composites could be obtained by means of SEM and EDS methods.

## 2. Materials and Methods

### 2.1. Materials and Composite Preparation

The cement used was PI 52.5 Portland cement. The relevant performance is shown in Table 1. In addition, purified graphene (initially 10–50 μm diameter, 1–5 layers, and 1.0–1.77 nm thickness; Hengqiu Graphene Technology Co., Ltd.) prepared by oxidation-reduction method was used. The graphene properties are presented in Table 2. Distilled water was applied for mixing of the grapheme/cement composites.

The water/cement ratio was set as 0.5, and 2.0 cm × 2.0 cm × 6.0 cm specimens were cast with 15 levels of graphene content, ranging from 0% to 2.5% by mass of cement. Before casting, graphene and cement were mixed in a commercial mixer with a rotating speed of 120 r/min for ten minutes. A specimen was filled with four pieces of conductive mesh electrodes (20 mm × 25 mm × 0.5 mm) with a spacing of 2 cm. Specimens were demolded after 24 h and then cured in a room under the condition of 95% RH and 25 °C for a month.

### 2.2. Electrical and Electrochemical Tests of Cement/Graphene Composites

**Figure 1:**
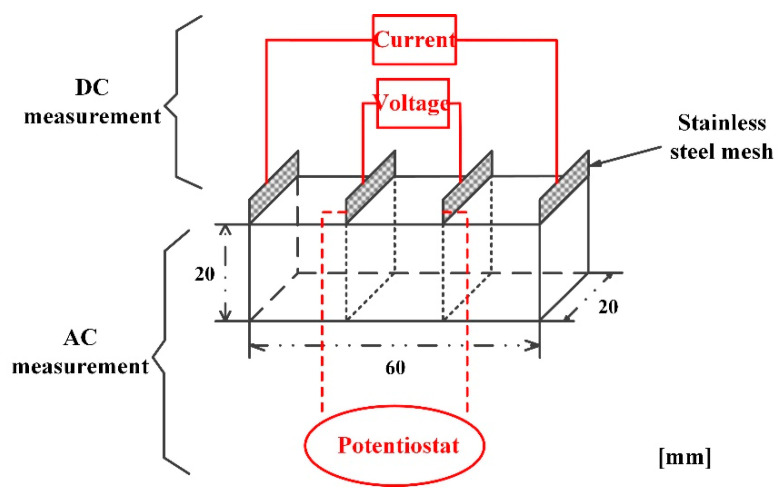
DC and AC measurements configuration.

The specimens were dried in a drying oven at 45 °C for 2 days in order to ensure completely dried specimens. Then, the specimen was conducted with direct current (DC) as well as alternating current (AC) measurements. It should be pointed out that the DC measurement was performed by means of the four-probe method. Moreover, the AC measurement was carried out using a Princeton Applied Research (PAR) START 2273 Potentiostat with a sinusoidal potential perturbation of 10 mV at the open circuit potentials and a frequency in the range from 10 mHz to 100 KHz. A detailed introduction concerning these two measurements is shown in Figure 1.

The electrical resistivity (ρ) is used to evaluate the composite’s electrical performance because the resistance (*R*) depends on the geometry of specimens, as expressed by Equation (1):

(1) $$\rho =\frac{R\cdot A}{L}$$

where A is the cross-sectional area of a specimen, and L is the length of specimen. Furthermore, the electrical conductivity (σ) of specimens is the reciprocal of their resistivity, as shown by Equation (2):

(2) $$\sigma =\frac{1}{\rho }$$

Meanwhile, the capacitance ability of the graphene/cement composites was analyzed based on the EIS results by means of the equivalent circuit model (ECM).

### 2.3. Microscale Characterization of Graphene/Cement Composites

A technique using scanning electron microscopy (SEM, Hitachi S4800, Hitachi, Japan) equipped with energy-dispersive spectrometers (EDS) was used to investigate the micro-morphology and element composition of the graphene/cement composites. Small-cored samples (approximately 2 mm × 2 mm × 0.5 mm) were collected from specimens and then used for SEM observation. These small-cored samples were immersed into a 95 vol% ethanol solution to stop hydration and then dried in an oven at 45 °C for 3 days. The surface of small-cored samples was covered with a thin gold layer before SEM observation.

## 3. Results and Discussion

### 3.1. Electrical Conductivity Measured by DC Measurement

**Figure 2:**
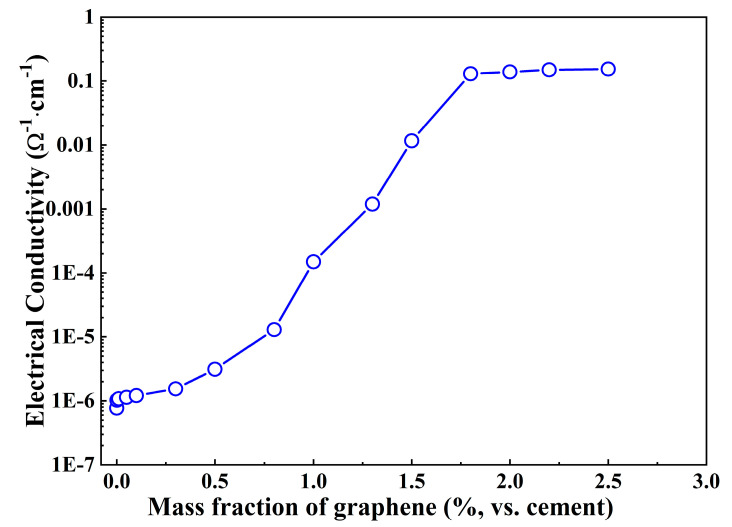
Conductivity vs. graphene content for graphene/cement composites obtained by DC measurement.

The electrical resistivity and conductivity values of the graphene/cement composites determined by direct current (DC) measurements versus the graphene content are plotted in Figure 2. Generally, the electrical resistivity value of the composites drops with the increase of the graphene content. In other words, the electrical conductivity increases with increasing graphene content. In the cases of plain cement paste and low graphene content, the electrical conductivity value of the specimen is in the order of 10^−6^, and this value grows slowly with the increase of graphene content. However, an abrupt increase of conductivity value has been observed once the graphene content exceeds 0.5% vs. cement. The conductivity changes by several orders of magnitude when the graphene content is more than 1.8% vs. cement. It is apparent that the composite’s conductivity is dependent on the graphene content.

Even though the DC measurement technique has some advantages, such as quick response and easy operation in measurement of the conductivity of composites, polarization has a heavy influence on the accuracy of results [11]. Therefore, the alternating current (AC) technique has been suggested for investigating the resistivity of the cement-based materials.

### 3.2. Electrical Conductivity Measured by AC Method

**Figure 3:**
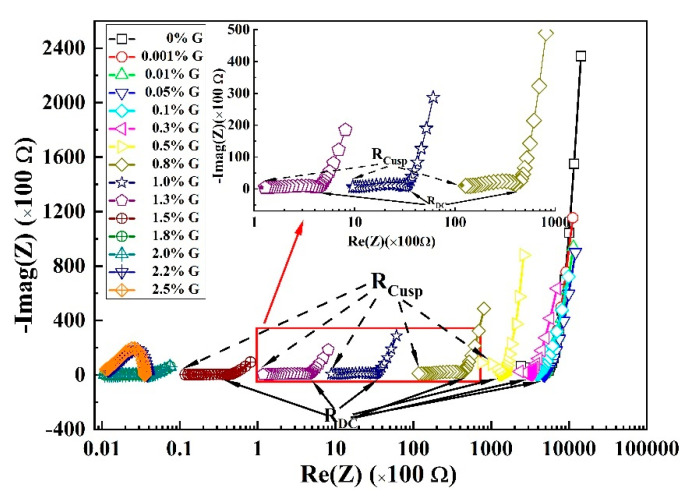
Nyquist curves obtained by AC measurement for graphene/cement composites with various graphene contents.

Figure 3 shows the AC measurement plots for the graphene/cement composites. Nyquist curves contain two arcs, namely the high-frequency and low-frequency arcs, respectively. Graphene particles behave as insulators at low frequencies, which have negligible effects on composites’ electrical conductivity. In contrast, displacement currents shorten graphene particles’ double layers at high frequencies, making them conductive (i.e., graphene particles act like short-circuit currents). Therefore, the significant decrease of electrical resistivity at the region of high frequencies is due to improvement in the conductivity by these conducting graphene particles. The low-frequency cusp corresponds to the DC resistance of cement/graphene composites, as shown by the solid symbols in Figure 3 [21,22]. Meanwhile, the high-frequency cusp, R_cusp_, referred to as the electrical resistivity at high frequency, is also highlighted in Figure 3.

**Figure 4:**
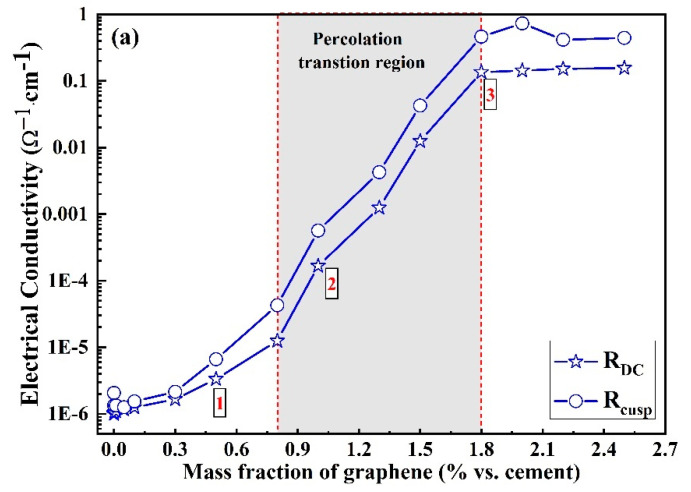

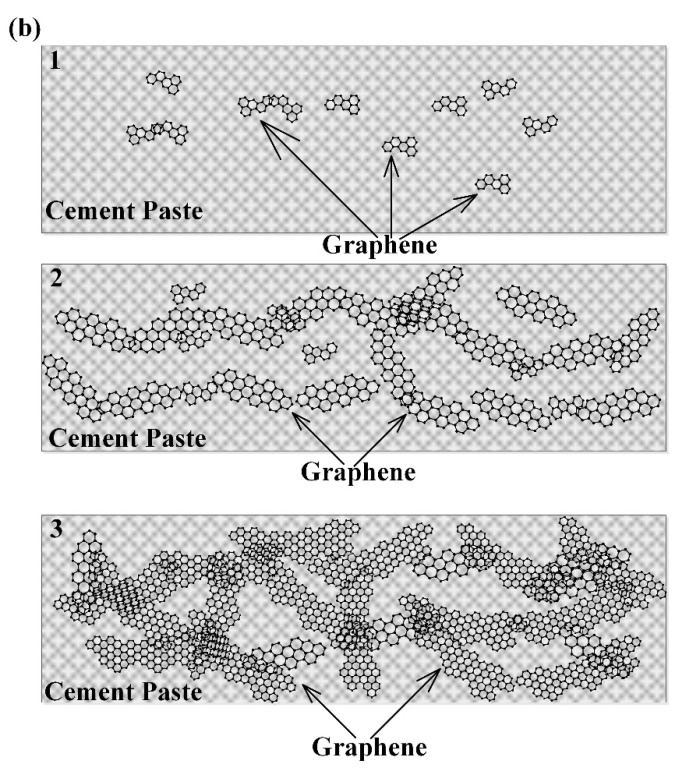
(**a**) Conductivities vs. graphene content for graphene/cement composites obtained by AC measurement. (**b**) Model of graphene distributed and interconnected in the composites.

Figure 4 indicates the evolution of electrical resistivity of the graphene/cement composites calculated based on the AC measurements with the graphene contents. In addition, a detailed model (Figure 4b) of graphene distribution and interconnection in the composite is also introduced in Figure 4 for different graphene contents. A similar S-shaped curve has been observed in Figure 4. Similar to the development of electrical conductivity values measured by DC technique, the electrical conductivity value of the composites increases with the increasing graphene content. Point 1 is representative of the situation of low graphene content: the graphene particles are distributed homogeneously in the composite, and there are few contacts between adjacent particles. Furthermore, point 2 represents the case in which larger graphene slices are produced when the graphene content reaches the threshold value (discussed below). Some local conductive networks have been formed due to these conductive particles being in touch with each other, which results in a dramatic increase of the electrical conductivity of the composites. However, beyond point 3, the conductivity of composites increases slightly with the increase of the graphene concentration because of the formation of the complete conductive networks throughout the composite. Therefore, a further increase of graphene content will not led to significant variation in the composite’s conductivity.

**Figure 5:**
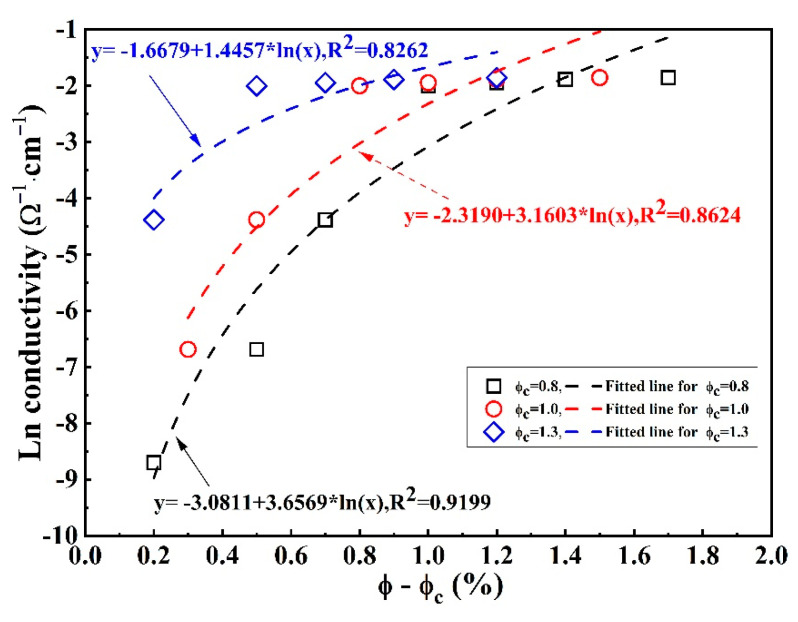
Conductivity as a function of $\varphi -\varphi_{c}$ for graphene/cement composites with different $\varphi_{c}$ values.

**Figure 6:**
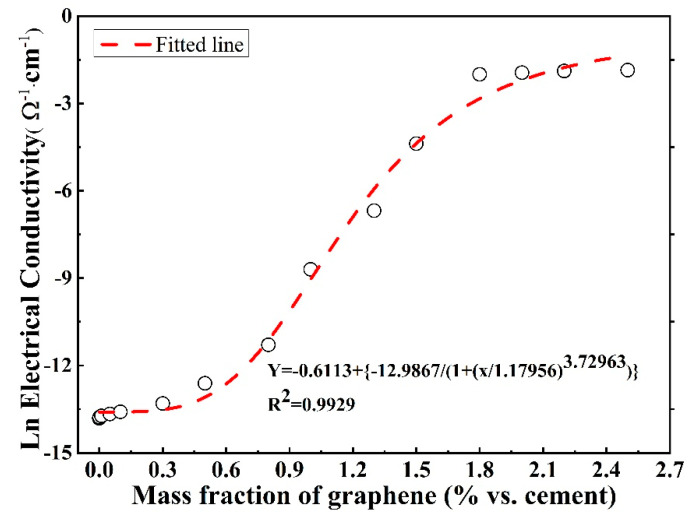
Fitted line for the conductivity vs. graphene content by means of logistic model.

There exists a narrow region in which the electrical conductivity increases quickly. This feature has been defined as the percolation phenomenon; based on this, this narrow region was usually defined as percolation transition zone $\varphi_{2}-\varphi_{1}$: $\varphi_{2}$ represents the largest graphene content of the percolation region, and $\varphi_{1}$ represents the minimum graphene content for entering the percolation region, as shown in Figure 4 [1]. In our investigation, the $\varphi_{2}$ and $\varphi_{1}$ values are determined as 1.8% and 0.8%, respectively. According to previous research [20], Equation (3) has been applied to characterize the relationship between the conductivity of the composite and the graphene content in the case of $\varphi >\varphi_{c}$:

(3) $$\sigma =a{\left(\varphi -\varphi_{c}\right)}^{t}$$

where t and a are two constants, φ is the graphene content, and the $\varphi_{c}$ value is defined as the threshold value ranging from $\varphi_{1}$ to $\varphi_{2}$. Therefore, three different $\varphi_{c}$ values ranging from $\varphi_{1}$ to $\varphi_{2}$ are selected to determine these relevant constants, as presented in Figure 5. In addition, these chosen $\varphi_{c}$ values and corresponding fitted equations are shown in Table 3. The a value seems to be larger with the increase of the threshold value because a is related to the conductivity of the conducting inclusions according to [23]. On the contrary, this t value decreases with increasing threshold value, although previous research reported that t was a constant value of 2 [24]. In addition, the effectiveness of the model can be evaluated through the application of the correlated $R^{2}$ value. It can be clearly determined that the deviation of the curve for the threshold value of 0.8% graphene content is much less than the others. However, this equation has some obvious limitations, such as it being difficult to determine the $\varphi_{c}$ value and the domain of the definition of this equation not including the graphene contents that are lower than the $\varphi_{c}$ value. Thus, there is an urgent need to discover a new model or equation by which the comprehensive characterization of the relationship between the conductivity and the whole graphene contents can be realized. We found that it is very appropriate to investigate the relationship between the conductivity and the graphene content by means of the logistic model, which has been usually applied to describe the S-shaped curve. The expression form of logistic model is as follows:

(4) $$y=A_{2}+\frac{A_{1}-A_{2}}{1+{\left(\frac{x}{x_{0}}\right)}^{p}}$$

where p, $A_{1}$ and $A_{2}$ are constants, $x_{0}$ is critical graphene concentration in the logistic model, y is the conductivity, and x is the graphene content. Therefore, the relevant fitted line for the correlation between the conductivity and the graphene content based on Equation (4) is presented in Figure 6, and the obtained equation is as follows:

(5) $$Ln\left(\sigma \right)=-0.61113+\frac{-12.9867}{1+{\left(\frac{\varphi }{1.17956}\right)}^{3.72963}}$$

This equation shows good agreement because the correlation coefficient for this model is as high as 0.9929. It accurately describes the relationship between the conductivity of composites and graphene concentration ranging from 0.001% to 2.5 % with respect to the mass of cement. In addition, it can precisely predict the evolution of the conductivity of composites with the graphene concentration. Some extra graphene contents in the graphene/cement composites and relevant conductivities are presented in Table 4; these predicted conductivities by means of Equation (5) are also listed in Table 4. The relative error (*RE*) is introduced to evaluate the difference between the measured (*M*) and predicted (*P*) values, which is expressed by Equation (6):

(6) $$RE=\frac{\left|P-M\right|}{M}\times 100\%$$

As shown in Table 4, the *RE* values for these chosen graphene concentrations are lower than 10%, which indicates that the predicted values calculated by Equation (5) have a small deviation compared to those measured values. This logistic model is recommended by the authors to characterize the relationship between the conductivity and the content of conductive materials such as steel powder, graphite powder, carbon fiber, carbon nanotube, and graphene in the composites.

### 3.3. Evaluation of Contribution of Graphene Particles in High Frequency

An “intrinsic conductivity” method has been introduced by Wansom [21,22] to investigate the micro-structure of cement-based conductive particle composites. In a dilute regime, assuming completely irregular distribution of graphene particles, the effective conductivity of the composites, $\sigma_{composite}$, containing the conductivity of graphene particles, $\sigma_{graphene}$, and mass fraction, ω, suspended in the conductivity of the matrix, $\sigma_{matrix}$, is presented in the following [21,22]:

(7) $$\frac{\sigma_{composite}}{\sigma_{matrix}}=1+{\left[\sigma \right]}_{\Delta }\omega +0\omega^{2}$$

where Δ is the ratio of the graphene conductivity to the matrix conductivity, and [σ] is the intrinsic conductivity. Note that for conductive graphene particles, the ratio between the graphene (10^3^ Ω^−1^·m^−1^) and plain cement paste (about 10^−6^ Ω^−1^·m^−1^) conductivities is near to infinity (i.e., Δ→∞). In addition, the first-order coefficient of ω functions as the “intrinsic conductivity” and can be obtained for any aspect ratio of graphene particles. The higher order terms should be neglected in the case of a dilute situation.

In addition, the ratio of the conductivity of composites (when the graphene particles are conducting at high frequency) to the conductivity of matrix is associated with the resistances in the EIS measurements given by:

(8) $$\frac{\sigma_{composite}}{\sigma_{matrix}}=\frac{R_{matrix}}{R_{composite}}=\frac{R_{DC}}{R_{cusp}}$$

where $R_{DC}$ value refers to the resistance at low frequency of the plain cement paste, and $R_{cusp}$ the resistance at high frequency for the graphene/cement composites.

Therefore, combining Equations (7) and (8) and rearranging yields:

(9) $$\frac{R_{DC}}{R_{cusp}}-1={\left[\sigma \right]}_{\Delta }\omega$$

Equation (9) indicates the whole contribution of graphene particles to the conductivity of graphene/cement composites. However, it must be pointed out that graphene dispersion issues, including orientation, coarse-scale isolation, and local aggregation, may be much too difficult to quantify for graphene/cement composites.

### 3.4. SEM Analysis

**Figure 7:**
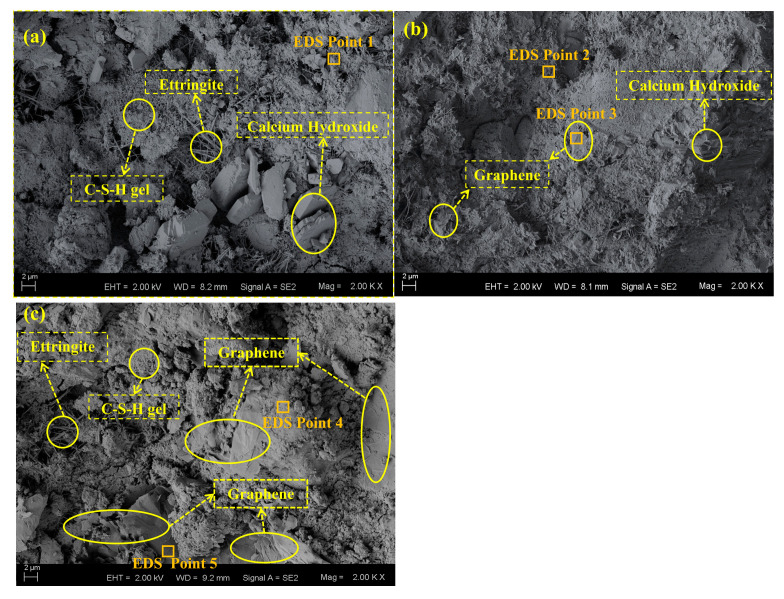
SEM graphs of composites (**a**) without graphene, (**b**) 0.8% graphene content, and (**c**) 1.8% graphene content.

Figure 7 indicates the micromorphology of graphene/cement composites without and with two graphene additions (0.8% and 1.8%). In the micromorphology of plain cement paste (Figure 7a), cement hydration products such as C-S-H gel, ettringite, and calcium hydroxide are clearly observed. Figure 7b exhibits the morphology of low graphene content (i.e., the content below the threshold value); graphene slices are distributed homogeneously in the cement pastes, and there are few contacts between the adjacent graphene particles, corresponding to the diagram in Figure 4b. These adjacent particles produce little influence on the improvement of the conductivity of composites. However, as the graphene content reaches the threshold value, graphene slices start to connect with each other to generate a conductive network, which results in the agglomeration of graphene slices, as clearly presented in Figure 7c. Compared to the graphene slices in Figure 7b, the slices in Figure 7c have larger sizes. In addition, many contacts between these graphene slices in c have also been formed, which can be seen vividly in Figure 4b. These micro-morphologies of graphene/cement composites based on the SEM technique clearly indicate the growth of the graphene slices with the increase of graphene concentration.

EDS analysis results of the chosen points in Figure 7 are also presented in Table 5. It should be pointed out that only five elements (i.e., carbon (C), oxygen (O), calcium (Ca), silicon (Si) and aluminum (Al)) were taken into account in this analysis. The results of point 1, 2, and 4 represent the element composition of cement hydration products in composites with different graphene contents, which show a high degree of similarity. The main elements in cement hydration products consist of Ca and O elements, followed by Si element. Small amount of C and Al elements are also detected. Differing from the cement hydration product, the element compositions of the graphene slices in composites are presented as point 3 and 5 in Table 5. Only one primary element (i.e., C element) has been found, as the mass fraction of C exceeds 80%. In other words, the results also prove that the slice in the composites is graphene because there is only C element in the composition of graphene. Previous analyses have shown that a low content of C element is detected in the cement hydration product. Through the EDS technique, the element compositions for the chosen places in the graphene/cement composites can be obtained, by which graphene slices can be distinguished from the cement hydration products.

## 4. Conclusions

This work measured the electrical conductivity of graphene/cement composites with different graphene additions by means of DC and AC methods. Through comparison, the conductivity of graphene/cement composites measured by AC technique is more accurate than that of DC due to the little influence of polarization.

There exists a percolation transition region $\varphi_{2}-\varphi_{1}$ (the $\varphi_{2}$ and $\varphi_{1}$ values are determined as 0.8% and 1.8%, respectively) in the S-shaped curve, which is used to describe the conductivity of composites with the addition of graphene. The logistic model has been recommended to characterize the relation between the conductivity of composites and graphene concentration ranging from 0.001% to 2.5 % vs. cement.

The micromorphology clearly indicates a complete conductive network as well as agglomeration of graphene slices when the graphene content reaches the threshold value. In addition, graphene slices can be distinguished from cement hydration products by their special morphology and elemental quantification through EDS technique. As far as we are concerned, it is promising to use graphene/cement composites as smart materials.

## Figures and Tables

**Table 1:** Oxide compositions of cement (%).

	SiO_2_	Al_2_O_3_	CaO	FeO	MgO	K_2_O	Na_2_O	TiO_2_	MnO	Loss
OPC	22.30	4.95	64.23	3.13	1.58	0.62	0.13	1.10	0.13	2.35

**Table 2:** Property of graphene.

Purity	Thickness	Diameter	Layers	Single Rate	Specific Surface Area
(wt.%)	(nm)	(μm)		(%)	(m^2^·g^−1^)
>90	1.0–1.77	10–50	1–5	>30	360–450

**Table 3:** Percolation threshold and corresponding fitted parameters and equations.

Threshold Value (%)	a	t	Fitted Equation	R^2^
0.8	0.0459	3.6569	$\sigma =0.0459{\left(\varphi -0.8\right)}^{3.6569}$	0.9199
1.0	0.0983	3.1603	$\sigma =0.0983{\left(\varphi -1.0\right)}^{3.1603}$	0.8624
1.3	0.1886	1.4457	$\;\sigma =0.1886{\left(\varphi -1.3\right)}^{1.4457}$	0.7630

**Table 4:** Measured and predicted conductivities for some extra graphene contents.

Graphene Content	Ln Measured Conductivity	Ln Predicted Conductivity	Relative Error
(%)	(Ω^−1^·m^−1^)	(Ω^−1^·m^−1^)	(%)
0.03	−13.7188	−13.5978	−0.8820
0.08	−13.6477	−13.5972	0.3700
0.20	−12.9234	−13.5805	5.0845
0.70	−11.4578	−11.9748	4.5122
1.20	−6.6987	−6.8965	2.9528
1.60	−3.8671	−3.7651	2.6376
2.10	−2.0134	−1.9645	2.4287
2.70	−1.0988	−1.1771	7.1259
3.00	−0.9198	−0.9986	8.5670

**Table 5:** Element compositions based on the EDS analysis.

Point	C (%)	O (%)	Ca (%)	Si (%)	Al (%)
1	4.71	50.29	34.41	9.88	0.71
2	3.41	49.29	35.88	10.61	0.81
3	82.69	12.50	2.87	1.54	0.40
4	7.96	48.45	33.46	8.97	1.16
5	85.69	9.50	3.15	1.38	0.28

## Data Availability

The data presented in this study are available on request from the corresponding author. The data are not publicly available due to privacy.
